# Chicago supermarket data and food access analytics in census tract shapefiles for 2007–2014

**DOI:** 10.1016/j.dib.2018.11.014

**Published:** 2018-11-06

**Authors:** Marynia Kolak, Michelle Bradley, Daniel Block, Lindsay Pool, Gaurang Garg, Chrissy Kelly Toman, Kyle Boatright, Dawid Lipiszko, Julia Koschinsky, Kiarri Kershaw, Mercedes Carnethon, Tamara Isakova, Myles Wolf

**Affiliations:** a Center for Translational Metabolism and Health, Institute of Public Health & Medicine, Northwestern University, 633N. St. Clair, 18th Floor, Chicago, IL 60611, USA; b Center for Spatial Data Science, Division of Social Sciences, University of Chicago, 5735 S Ellis Ave, Room 232, Chicago, IL 60637, USA; c Geography Program, Chicago State University, 9501S. King Drive, Chicago, IL 60628, USA; d Division of Nephrology and Hypertension, Feinberg School of Medicine, Northwestern University, 251 East Huron Street, Galter Suite 3-150, Chicago, IL 60611, USA

## Abstract

Longitudinal analysis of supermarkets over time is essential to understanding the dynamics of foodscape environments for healthy living. Supermarkets for 2007, 2011, and 2014 for the City of Chicago were curated and further validated. The average distance to all supermarkets along the street network was constructed for each resident-populated census tract. These analytic results were generated with GIS software and stored as spatially enabled data files, facilitating further research and analysis. The data presented in this article are related to the research article entitled “Urban foodscape trends: Disparities in healthy food access in Chicago, 2007–2014” (Kolak et al., 2018).

**Specifications table**

**Table:** 

Subject area	Social Science
More specific subject area	Public Health, Geography
Type of data	Spatial data files (ESRI shapefile) and supporting figures
How data was acquired	ArcMap was used to generate the shapefiles and associated results
Data format	Raw, analyzed
Experimental factors	Supermarket data for the City of Chicago was curated and validated for 2007, 2011, and 2014. A cost distance analysis was implemented to calculate food market accessibility for each corresponding year. Raw and adjusted access measures are reported by census tract.
Experimental features	The average distance along the street network of residential areas to nearest supermarkets was calculated and averaged for each resident-populated census tract for each year. An adjusted food access measure accounts for underlying tract population.
Data source location	Chicago, Illinois
Data accessibility	Data is made available with this article.
Related research article	Kolak M, Bradley M, Block DR, Pool L, Garg G, Toman CK, Boatright K, Lipiszko D, Koschinsky J, Kershaw K, Carnethon M. Urban foodscape trends: Disparities in healthy food access in Chicago, 2007–2014. Health and Place. 2018 Jul 1;52:231–239 [1]

**Value of the data**

• This series of food access data is unique in providing only validated supermarkets that met both classification requirements and confirmed field verification for three time periods.
• Datasets provided showing supermarkets in Chicago over time are invaluable for researchers seeking to better understand impacts from changes in food access.
• Data tracking mean network distance to supermarkets for each census tract in Chicago serves as a new benchmark to measure localized food access for research and health workers.

## 1 Data

The supermarket dataset details locations of chain and independent supermarkets in the Chicago area in 2007, 2011, and 2014. This dataset is a collection comprised of four ESRI shapefiles: first, three files that contain point locations for each supermarket, for each year. The tract-level dataset is a single ESRI shapefile of polygon data corresponding to Chicago census tract boundaries, with additional details for each of the 791 tracts used in the original study. Details include: (1) Mean Distance to all Supermarkets for each tract, and (2) Population in 2012, used for calculation of an adjusted measure. The data view, without spatial information, can likewise be extracted from the associated.dbf file.

## 2 Experimental design, materials and methods

**Fig. 1:**
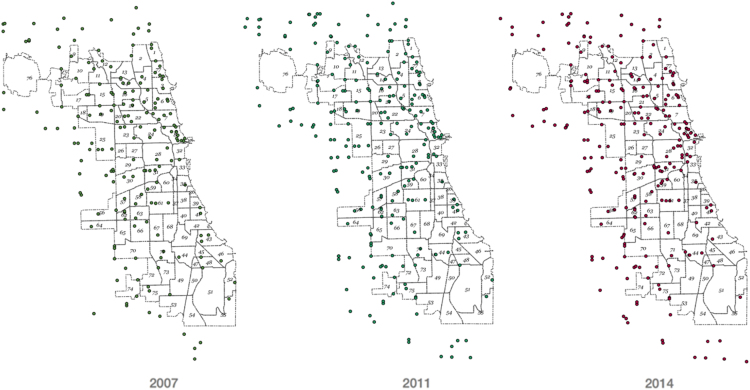
Supermarket Locations across Chicago for years 2007, 2011, and 2014. Locations bordering Chicago included. Chicago community area boundaries shown with community area identification number.

**Fig. 2:**
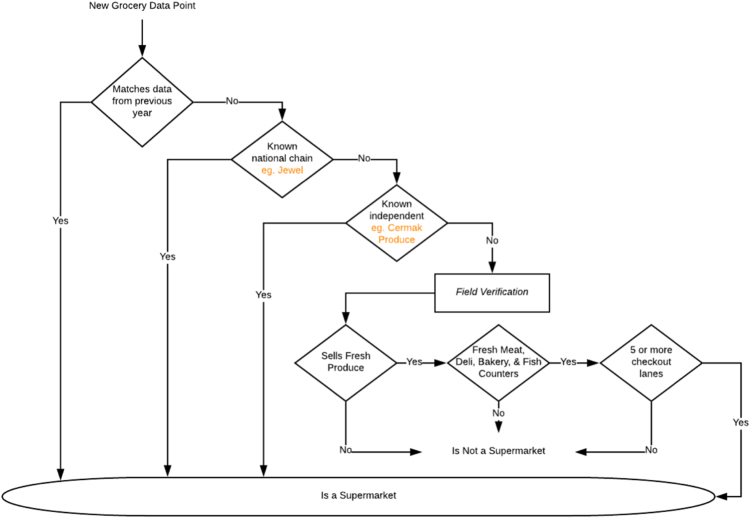
Supermarket Locations across Chicago for years 2007, 2011, and 2014. Locations bordering Chicago included. Chicago community area boundaries shown with community area identification number.

The supermarket dataset was curated from public and private sources, cross-referenced, and further validated with in-person field verification. More details on the data process, decision tree used to classify stores, and data design are available in Kolak et al. [1]. Data collection and auditing was performed during each of the three study years using Block and Kouba field methodology [2], building on the original data [7]. Supermarket locations for each year in the Chicago area are visible in Fig. 1. A flow diagram of the process we used to define supermarket status is provided in Fig. 2.

**Fig. 3:**
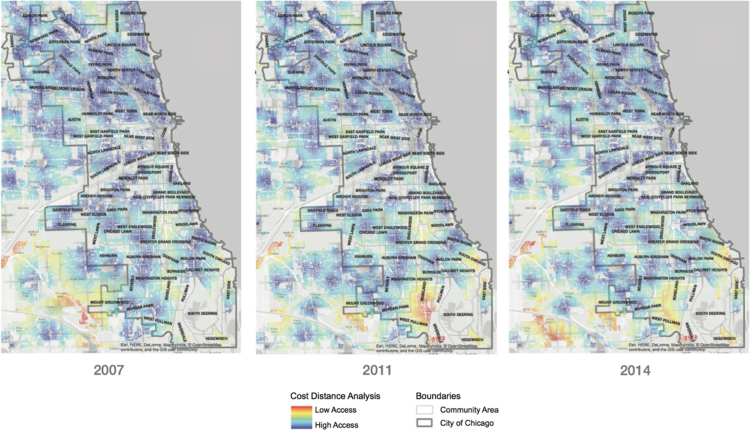
Cost distance calculations on residential and mixed-use street networks for each year of analysis.

The average distance from residential areas to the nearest supermarket, along the street network, was calculated for the study area using potential access or cost distance methodology [2], [3], [4], [5], [6]. To accomplish this, we converted the street network of Chicago and surrounding counties from vector (graph representation) to raster (pixelated representation) format to generate a fine-resolution grid map accurate to the nearest ten feet (see Fig. 3). Non-residential locations were removed based on zoning information provided by the Chicago Metropolitan Association.

**Fig. 4:**
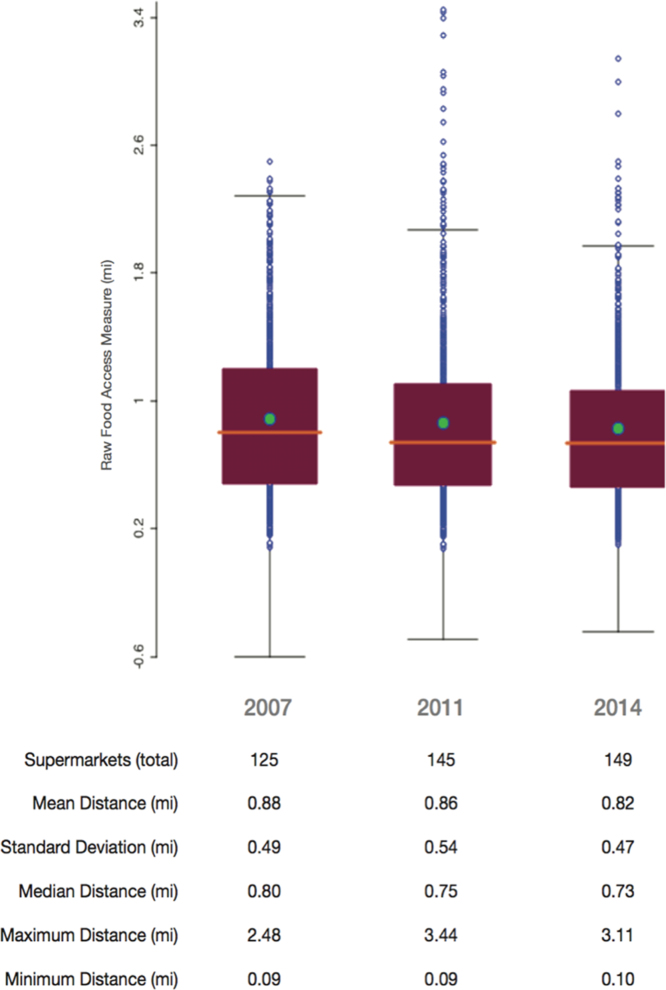
Box plot representation of the distribution of the average raw food access index by each year of analysis.

The average network distance to supermarkets was then averaged for each census tract in the Chicago area that had a residential population (*n* = 791). The distributions of the average raw food access index of the 791 resident-populated census tracts are presented in Fig. 4 for each year of analysis. This raw measure is recorded in the tract shapefile in both feet and miles. An adjusted measure represents the standardized average by population, per tract.

**Fig. 5:**
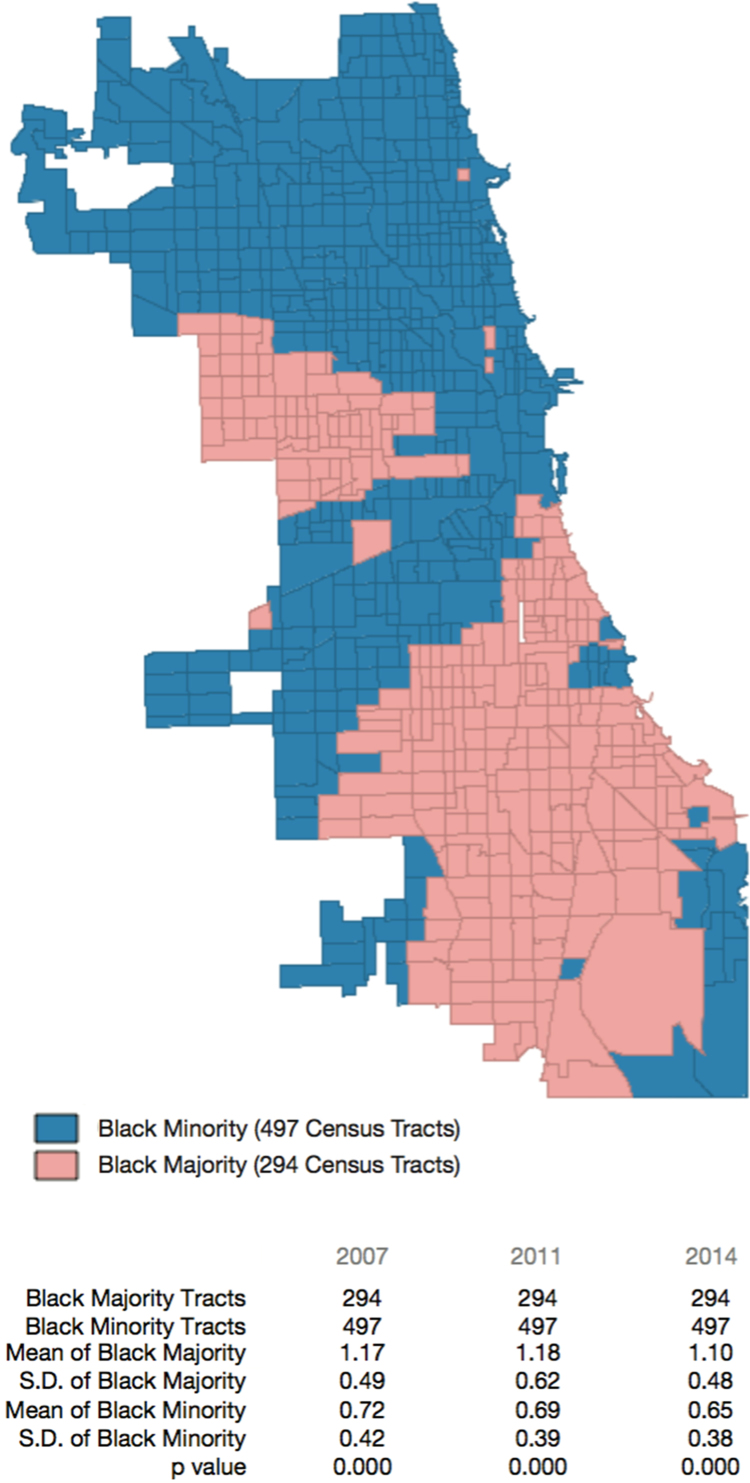
Map of the City of Chicago that color codes census tracts according to whether or not they include a majority black population. Statistics comparing the average raw food access index between black majority and minority census tracts are inset.

To evaluate food access between segregated residents in Chicago, we categorized census tracts as having or not having a majority (>50%) population of Black residents and compared the raw food access index between these census tracts in each year, using a comparison of means in GeoDa open source software (Fig. 5). Additional analysis was presented in Kolak et al. [1].
